# Epilepsy

**Published:** 1891-01

**Authors:** 


### Book Reviews.

EPILEPSY: Its Pathology and Treatment. Being the Essay to which was awarded a prize of 4,000 francs by the Academie Royale de Médécine de Belgique, December 31, 1889. By HOBART AMORY HARE, M. D., B. Sc., Clinical Professor of the Diseases of Children and Demonstrator of Therapeutics in the University of Pennsylvania; Physician to St. Agnes' Hospital, and to the Children's Dispensary of the Children's Hospital, etc. No. 7 in the Physician's and Student's Ready Reference Series. Philadelphia and London; p. 25. Cloth, $1.25. F. A. Davis. 1890.

That this is the Belgian Prize Essay, gives a peculiar interest in reading this monograph.

As a complete work it leaves nothing to be desired. The different types of Epilepsy are all carefully considered, and the arguments of different observers on mooted points are carefully weighed and judged.

We must confess that we looked to the section on prognosis in hopes that we might feel justified in taking brighter views of this disease.

In answer to the question, Will there be any progress toward an improvement? the author says:

Unfortunately, the reply ought not, in any case of the idiopathic form, to be favorable, even for ultimate improvement, for the experience in the past of every large practitioner has been that cures rarely occur. . . . Too much encouragement should not be held out in he use of drugs ; but this should not be impressed upon the patient's mind, since it is come to render him careless in taking the remedies prescribed.

Of syphilitic epilepsy he says :

In regard to this form, it may be asserted, with no fear of contradiction, that it can, in the majority of cases, be cured, and in nearly all cases improved.

Under the head of treatment we find that he places bromide of potassium at the head of the list, and calls it the most useful drug in use today.

Of antifebrin he says : "It stands in the foremost rank, and bids fair in some instances to rival the bromides." He uses eight grains three times daily. The other drugs used in this disease are mentioned and their merits discussed.

Of Féré's method of applying the cautery to the scalp in cases of epilepsy with hemiplegia, we were surprised to find no mention, as good results have been obtained by him.

We can heartily recommend this book to those who wish to make a thorough study of this disease. J. W. P.

